# Effect of Phosphoglucosamine Mutase on Biofilm Formation and Antimicrobial Susceptibilities in *M. smegmatis glmM* Gene Knockdown Strain

**DOI:** 10.1371/journal.pone.0061589

**Published:** 2013-04-10

**Authors:** Jian Kang, Liming Xu, Shufeng Yang, Wendan Yu, Shuo Liu, Yi Xin, Yufang Ma

**Affiliations:** 1 Department of Biochemistry and Molecular Biology, Dalian Medical University, Dalian, China; 2 Department of Biotechnology, Dalian Medical University, Dalian, China; 3 Liaoning Provincial Core Lab of Glycobiology and Glycoengineering, Dalian, China; Infectious Disease Research Institute, United States of America

## Abstract

UDP-N-acetylglucosamine (UDP-GlcNAc) is a direct glycosyl donor of linker unit (L-Rhamnose-D-GlcNAc) and an essential precursor of peptidoglycan in mycobacteria. Phosphoglucosamine mutase (GlmM) is involved in the formation of glucosamine-1-phosphate from glucosamine-6-phosphate, the second step in UDP-GlcNAc biosynthetic pathway. We have demonstrated that GlmM protein is essential for the growth of *M. smegmatis*. To facilitate the analysis of the GlmM protein function in mycobacteria, a tetracycline inducible *M. smegmatis glmM* gene knockdown strain was constructed by using an antisense RNA technology. After induction with 20 ng/ml tetracycline, the expression of GlmM protein in *glmM* gene knockdown strain was significantly decreased, resulting in a decline of cell growth. The morphological changes of *glmM* gene knockdown strain induced with 20 ng/ml tetracycline have been observed by scanning electron microscope and transmission electron microscope. Furthermore, insufficient GlmM protein reduced the biofilm formation and increased the sensitivity to isoniazid and ethambutol in *M. smegmatis*, indicating that GlmM protein had effect on the biofilm formation and the senstivity to some anti-tuberculosis drugs targeting the cell wall. These results provide a new insight on GlmM functions in mycobacteria, suggesting that GlmM could be a potential target for development of new anti-tuberculosis drug.

## Introduction

Tuberculosis, an infectious disease caused by *M. tuberculosis*, remains an important problem the world is facing due to the alarming increase of people living with HIV and the emergence of multi- and extensively drug-resistant strains [1], [2]. Thus, new drugs against this stubborn disease are urgently needed. As far as we know, *M. tuberculosis* has a unique and complex cell wall structure which plays essential roles in controlling bacterial growth, surviving in the infected host, and the immunologic response [3], [4], [5]. Due to the importance of cell wall to *M. tuberculosis* viability, the enzymes involved in the biosynthesis of cell wall are potential targets for anti-tuberculosis drugs.

The cell wall architecture of mycobacteria amounts to a massive ‘‘core’’ comprised of peptidoglycan covalently attached via a linker unit (L-Rhamnose-D-GlcNAc) to a linear galactofuran, in turn attached to several strands of a highly branched arabinofuran, in turn attached to mycolic acids [4]. UDP-N-acetylglucosamine (UDP-GlcNAc) is a directly glycosyl donor of linker unit, which is involved in the attachment of galactofuran and peptidoglycan, and it is also the precursor of peptidoglycan [6], [7]. In bacteria, UDP-GlcNAc is synthesized from the glycolytic intermediate D-fructose-6-phosphate by four successive reactions catalyzed by three enzymes: glucosamine-6-phosphate synthase (GlmS), phosphoglucosamine mutase (GlmM) and the bifunctional enzyme glucosamine-1-phosphate acetyltransferase/N-acetylglucosamine-1-phosphate uridyltransferase (GlmU) [6], [8], [9], [10], [11]. Interestingly, GlmM protein which catalyses the interconversion of glucosamine-6-phosphate and glucosamine-1-phosphate is unique to prokaryotes.

*M. smegmatis*, a fast growing and non-pathogenic species which was homologous with *M. tuberculosis*, was commonly used as a surrogate host for pathogenic *M. tuberculosis*. We had recently constructed a conditional *M. smegmatis glmM* gene knockout strain and confirmed that *glmM* gene was essential for the growth of *M. smegmatis* [12], indicating that GlmM could be used as a potential target of anti-tuberculosis drugs. Therefore, the biological functions of GlmM enzyme in mycobacteria need to be further illustrated.

Inducible gene expression systems are powerful tools for studying gene functions in bacteria, especially the essential genes which would lead a lethal phenotype when they were inactivated. Blokpoel *et al* had reported a tetracycline inducible system for use in mycobacteria and the application of this system for conditional gene silencing had been demonstrated [13]. The tetracycline inducible express vector pMind is a mycobacterial shuttle plasmid which has been sub-cloned with a *tetRO* operator-repressor region from *Corynebacterium glutamicum TetZ* [13]. The target gene is inversely inserted into the downstream of *tetRO* and the plasmid will express antisense RNA in the response of tetracycline [13]. The expressed antisense RNA will base pair with the target mRNA, thereby preventing ribosome binding and target protein translation [14]. This provided us a genetic approach to characterize essential genes whose deletion would cause death of the bacteria.

To gain insights into the *glmM* functions in mycobacteria, a tetracycline inducible antisense gene expression system was constructed to down regulate the *glmM* gene in *M. smegmatis*. The cellular growth of *M. smegmatis glmM* gene knockdown strain was monitored by optical density and colony forming units. The GlmM protein expression in *M. smegmatis glmM* gene knockdown strain was detected by anti-GlmM polyclonal antibody we prepared in our lab. The morphology of *M. smegmatis glmM* gene knockdown strain was also observed by scanning electron microscope (SEM) and transmission electron microscope (TEM). In addition, the biofilm formation and antimicrobial susceptibility of *glmM* gene knockdown strain were detected to further elucidate the biological roles of GlmM protein in *M. smegmatis*.

## Materials and Methods

### Bacterial strains, plasmids, and culture conditions

The bacterial strains and plasmids used in this study are listed in Table 1. NovaBlue strain was grown in Luria-Bertani (LB) medium at 37°C routinely. *M. smegmatis* mc^2^155 strain was grown in LB broth containing 0.05% Tween 80 or on LB agar at 37°C routinely. *M. smegmatis* mc^2^155 was used for cloning *glmM* gene (420 bp) and constructing a *M. smegmatis glmM* gene knockdown strain. Antibiotics were added at the following final concentrations: ampicillin (Amp), 100 µg/ml; kanamycin (Kan), 50 µg/ml for *E. coli* and 25 µg/ml for *M. smegmatis*.

**Table 1:** Bacterial strains and plasmids used in this study.

Strains/Plasmids	Description	Source
**Strains**		
*E. coli* NovaBlue	Used for cloning and propagation of plasmids	Novagen
*M. smegmatis* mc^2^155	Used as a DNA template to amplify DNA fragment of *M. smegmatis glmM* (420 bp)	ATCC
*M. smegmatis* AS	*M. smegmatis* mc^2^155 carrying pMind-*glmM*-420-AS	This study
**Plasmids**		
pMD18-T	Carries *amp^R^* gene; used for cloning PCR product with A at 3′ ends	Takara
pMind	Carries *kan^R^* gene; used for expression of *glmM* antisense mRNA	[13]
pMD-*glmM*-420	The DNA fragment (420 bp) of *M. smegmatis glmM* was cloned to the EcoRV site of pMD18-T	This work
pMind-*glmM*-420-AS	The DNA fragment (420 bp) of *M. smegmatis glmM* in pMD-*glmM*-420 was cloned to the SpeI and NdeI sites of pMind	This work

### Ethics Statement

All animal experimental procedures were conducted in conformity with institutional guidelines for the care and use of laboratory animals in Dalian Medical University, and conformed to the National Institutes of Health Guide for Care and Use of Laboratory Animals. The protocol was approved by the Committee on the Ethics of Animal Experiments of Dalian Medical University. Permit Number: SYXK (Liao) 2008-0002.

### Construction of *glmM* gene knockdown strain, *M. smegmatis* AS

The DNA fragment (420 bp) of *M. smegmatis glmM* gene in 5′ end was amplified from *M. smegmatis* mc^2^155 genomic DNA by PCR using primers glmM1 (5′-TTCATATGGAGTTCCTCGATGCGGTC-3′, underlined is NdeI site) and glmM2 (5′-TTACTAGTATGGCTCGACTGTTCGGC-3′, underlined is SpeI site) respectively. The PCR product (420 bp) was ligated into pMD18-T vector to generate pMD-*glmM*-420 plasmid (Table 1) and confirmed by DNA sequencing. The DNA fragment (420 bp) of *glmM* was ligated into SpeI and NdeI sites of pMind yielding pMind-*glmM*-420-AS (Table 1).

M. smegmatis mc^2^155 electro-competent cells were prepared as described [15]. The pMind-*glmM*-420-AS plasmid was electroporated into mc^2^155 competent cells by Electroporator 2510 (Eppendorf), yielding a *glmM* gene knockdown strain, *M. smegmatis* AS.

### Growth of *M. smegmatis* AS strain

The cellular growth of *M. smegmatis* AS strain was monitored by optical density (OD) and colony forming units (CFU). One colony of *M. smegmatis* AS strain was inoculated into 5 ml of LB broth containing 0.05% Tween 80 and Kan at 37°C. When OD600 reached to 0.5, 10 µl of culture was inoculated into 20 ml LB broth containing 0.05% Tween 80 and Kan at 37°C. The tetracycline at different concentrations (0 ng/ml, 10 ng/ml, 20 ng/ml, 30 ng/ml and 50 ng/ml) was added into the cultures as inducer. The OD600 of the cultures was monitored at interval of 12 h. For CFU determinations (taken at the same time points), dilutions were spread on LB agar plates. The plates were incubated at 37°C before CFU were counted. The growth of wild type mc^2^155 strain with different concentrations of tetracycline was also performed as described.

### Preparation of polyclonal antibody against *M. smegmatis* GlmM protein

Anti-GlmM polyclonal antibody was prepared by immunizing method using purified *M. smegmatis* GlmM protein. *M. smegmatis* GlmM protein was purified as described previously [12]. The GlmM protein (100 µg) in 100 µl physiological saline was emulsified with 100 µl of the Freund's incomplete adjuvant (Thermo Scientific). Eight 8-weeks old female BALB/c mice housed in the Animal Center of Dalian Medical University were used for preparation of polyclonal antibody. Before inoculation of antigen, 500 µl of blood was collected from the orbital venous plexus of mice for preparing the pre-immunized-serum as a negative control. On the 1st, 10th, 17th day, the emulsified GlmM protein (100 µg) was injected intradermally on 3 parts of the mouse body. The blood was collected from the orbital venous plexus of mice on the 24th day. Blood samples were kept at 4°C for 4 h for coagulation. The anti-serum was separated by centrifugation at 4°C, 10000 g for 10 min and stored at −80°C. Western blot and ELISA was performed to evaluate the specificity and titers of produced polyclonal antibody.

### Expression of GlmM protein in *M. smegmatis* AS strain

Western blot was used to analyze the expression levels of GlmM protein in wild type mc^2^155 strain and *M. smegmatis* AS strain. *M. smegmatis* AS strain was grown in 100 ml LB broth containing 0.05% Tween 80 and Kan at 37°C. The tetracycline at different concentrations (0 ng/ml, 20 ng/ml) was added into the medium at the beginning of incubation. The cells were harvested after incubation for 36 h and resuspended in 3 ml lysis buffer (20 mM Tris-HCl, pH 8.0, 500 mM NaCl and 20% glycerol) with 1 mM phenylmethyl-sulphonyl fluoride followed by sonication. The samples were centrifuged at 20000 g for 30 min, and the supernatant was collected for Western blot analysis. The supernatants of wild type mc^2^155 strain induced with different concentrations of tetracycline (0 ng/ml, 20 ng/ml) were also collected as described.

The same amount of soluble protein in supernatant from different samples were run on 12% SDS-PAGE and transferred to a nitrocellulose membrane in blotting buffer (20 mM Tris-base, 150 mM glycine and 20% methanol). The membrane was blocked with 1% BSA in TBST buffer (10 mM Tris–HCl, pH 8.0, 150 mM NaCl, 0.05% Tween 20) and incubated with anti-GlmM polyclonal antibody at 1∶5000 dilution followed by washing with TBST. The membrane was incubated with antimouse-IgG-AP conjugate and the protein band was visualized in BCIP/NBT solution.

### Morphology of *M. smegmatis* AS strain

*M. smegmatis* AS strain induced with or without 20 ng/ml tetracycline was grown in 20 ml LB broth at 37°C. The cells were harvested after incubation for 36 h. The cell pellet was washed three times in the 0.1 M Phosphate buffer (pH 7.4) and fixed with 2.5% glutaraldehyde followed by fixation with 1% OsO4. The cells were then dehydrated in a graded series of ethanol (20, 40, 60, 70, 80, 90, and 100%). The cells for scanning electron microscopy (SEM) observation were critical point dried and applied to a silicon wafer slide and coated by gold [16]. The cells were then observed by using a JSM-6360 scanning electron microscope (JEOL). The cells for transmission electron microscope (TEM) observation were embedded in the Epon 812 embedding kit and cut into ultrathin sections. The sections were double-stained with uranyl acetate and lead nitrate and then observed using a JEM-2000EX TEM (JEOL). The SEM observations of wild type mc^2^155 strain induced with 20 ng/ml tetracycline were also performed as described.

### Determination of biofilm formation

Wild type mc^2^155 strain and *M. smegmatis* AS strain were inoculated in LB broth containing 0.05% Tween 80 at 37°C. When OD_600_ reached to 0.5, the cells were diluted 1∶1000 in M63 medium supplemented with 0.5% Casamino acids, 1 mM MgSO4 and 0.7 mM CaCl2. The tetracycline at different concentrations (0 ng/ml, 20 ng/ml) was also added into the medium. The experiment was carried out in triplicate. The culture of 150 µl was added to each well in a 96-well polystyrene microtiter plate. The plates were incubated at 30°C for 5 days without disturbance. The wells were rinsed three times with physiological saline, and 150 µl of 1% crystal violet was added for stain. The plates were incubated at room temperature for 15 min and rinsed with water three times. Quantitation of biofilm formation was performed by extracting crystal violet associated with biofilm by 95% ethanol and measuring OD570.

### Antimicrobial susceptibilities of *M. smegmatis* AS strain

The susceptibilities of wild type mc^2^155 strain and *M. smegmatis* AS strain to various antibiotics were determined by using the resazurin reduction microplate assay (REMA) method [17] with modification. Resazurin, a blue and nonfluorescent indicator dye, is converted to resorufin (pink and highly fluorescent) via the reduction reactions of metabolically active cells. The amount of fluorescence produced can be detected at 590 nm by a microplate reader, and it is proportional to the number of living cell.

Five first-line anti-tuberculosis drugs were tested: isoniazid (INH), rifampin (RMP), streptomycin (STR), ethambutol (EMB) and pyrazinamide (PZA). INH, STR, EMB and PZA were dissolved in sterilized water, and RMP was dissolved in methanol. Stock solutions were diluted in LB broth to two times of the maximal desired concentration. A sterile 96-well microtiter plate was used to perform MABA. Sterilized distilled water of 200 µl was added into all outer perimeter wells to prevent dehydration during incubation. LB broth (50 µl) was added into other wells in columns 3 to 10, and 50 µl of 2× drug solution was added into wells in columns 2 and 3. A serial 2-fold dilution of drug from columns 3 to 10 was performed directly in the plate. The wells in column 11 were filled with 50 µl LB broth served as drug-free controls. The final concentration ranges of drugs were as follows: INH 0.039–10 µg/ml, RMP 0.39–100 µg/ml, STR 0.05–12.5 µg/ml, EMB 0.01–2.5 µg/ml, PZA 0.002–0.5 mg/ml.

*M. smegmatis* AS strain was inoculated in LB broth containing 0.05% Tween 80 at 37°C. When OD_600_ reached to 0.5, the cells were diluted 1∶2000 in LB broth containing 0.05% Tween 80, and 50 µl dilutions with or without 20 ng/ml tetracycline were added to each well. The plates were covered and sealed with parafilm and incubated at 37°C. After 30 h of incubation, 100 µl of freshly prepared 1∶1 mixture of 10× Resazurin (Sigma) and 10% Tween 80 were added to each well and the plates were incubated for an additional 5 h at 37°C. A color change from blue to pink indicated cell growth, and the fluorescence of the resazurin metabolite resorufin was measured at OD_590_ by using Multiskan FC microplate reader (Thermo Scientific). The MIC was defined visually as the lowest drug concentration at which there was no color change from blue to pink and confirmed by the level of fluorescence measured by the microplate reader. The susceptibilities of wild type mc^2^155 strain to various antibiotics were also determined as described.

## Results

### Growth of *M. smegmatis* AS strain regulated by tetracycline was dose-dependent

**Figure 1:**
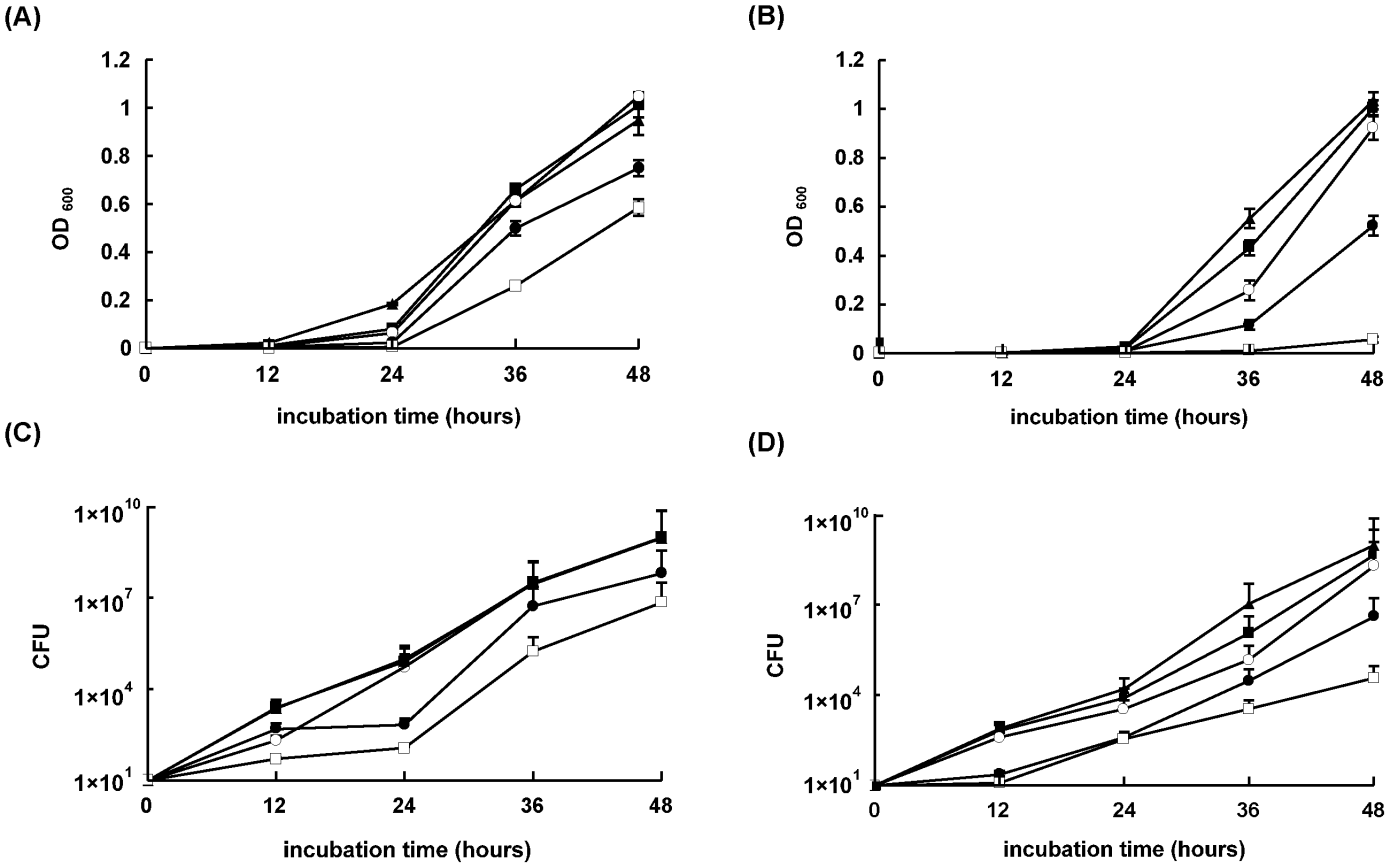
Bacterial growth of wild type mc^2^155 strain and *M. smegmatis* AS strain. The cultures were grown in LB broth containing 0.05% Tween 80 at 37°C with different concentration of tetracycline. The OD600 and CFU of the cultures were monitored at interval of 12 h. A. Growth curve of the wild type mc^2^155 strain; B. Growth curve of the *M. smegmatis* AS strain; C. CFU of the wild type mc^2^155 strain; D. CFU of the *M. smegmatis* AS strain. (▴) The cultures without tetracycline; (▪) The cultures induced with 10 ng/ml tetracycline; (○) The cultures induced with 20 ng/ml tetracycline; (•) The cultures induced with 30 ng/ml tetracycline; (□) The cultures induced with 50 ng/ml tetracycline. The values plotted are the mean value and standard deviation from triplicate experiments.

A dose response curve was established by inducing expression of *glmM* antisense RNA in *M. smegmatis* AS strain with different concentrations of tetracycline (Fig. 1B). The growth rates of the tetracycline-induced AS strain were reduced compared to those of the uninduced AS strain. With the increase of tetracycline concentration, the growth rates of *M. smegmatis* AS strain slowed down. The growth curve of the wild type mc^2^155 strain showed that induction by 20 ng/ml tetracycline would not affect normal growth of bacteria. When induced with 30 ng/ml tetracycline, the growth rate of wild type strain would decrease slightly (Fig. 1A). CFU determinations were taken at the same time points, and the results showed the same tendency as the OD_600_ value (Fig. 1C, 1D). Considering the induction efficiency of tetracycline and the influence of tetracycline to bacterial normal growth, 20 ng/ml tetracycline was used as inducer for the further experiments.

In addition, the OD_600_ value and CFU of *M. smegmatis* mc^2^155 carrying pMind were detected, and the result was consistent to the growth curve of wild type mc^2^155 strain (data not shown). The comparison of two growth curves of uninduced *M. smegmatis* AS strain and uninduced wild type mc^2^155 strain was also performed, and no significant difference was found (data not shown).

Interestingly, the growth of *M. smegmatis* AS strain presented the maximal inhibitory effect at 36 h. For this reason, the cultures induced for 36 h were collected for detecting GlmM protein expression and observing morphological changes.

### Expression of GlmM protein was dramatically decreased in *M. smegmatis* AS strain

**Figure 2:**
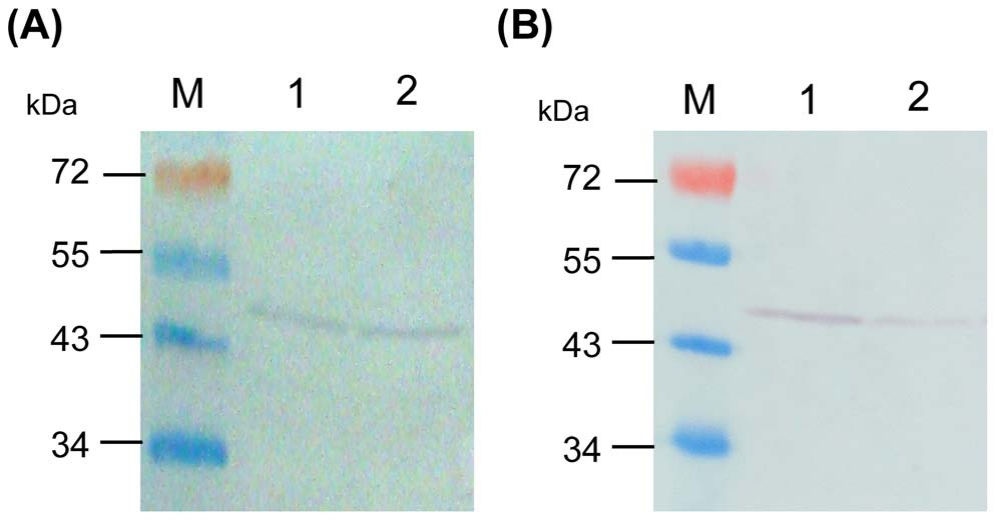
Expression of GlmM protein in wild type mc^2^155 strain and *M. smegmatis* AS strain. The expressions of *M. smegmatis* GlmM proteins in wild type mc^2^155 strain (A) and *M. smegmatis* AS strain (B) were detected by using anti-GlmM polyclonal antibody. The results showed that there were no obvious differences of GlmM protein expressions in wild type mc^2^155 strain with or without 20 ng/ml tetracycline. However, compared with uninduced *M. smegmatis* AS strain, the expression of GlmM protein was 70% decreased in *M. smegmatis* AS strain when induced with 20 ng/ml tetracycline. M. PageRuler prestained protein ladder; lane 1. GlmM proteins in uninduced cultures; lane 2. GlmM proteins in cultures induced with 20 ng/ml tetracycline.

The productions of GlmM protein in wild type mc^2^155 strain and *M. smegmatis* AS strain induced with or without 20 ng/ml tetracycline were detected by using Western blot. The results showed that the expression of GlmM protein was 70% decreased in *M. smegmatis* AS strain induced with 20 ng/ml tetracycline, compared to GlmM protein in uninduced *M. smegmatis* AS strain (Fig. 2B). However, no obvious differences of GlmM protein expression in wild type mc^2^155 strain with or without 20 ng/ml tetracycline were observed (Fig. 2A), and the expressions of GlmM protein in wild type mc^2^155 strain were similar to *M. smegmatis* AS uninduced strain. The results of Western blot demonstrated that the biosynthesis of GlmM protein could be inhibited effectively under the control of 20 ng/ml tetracycline in *M. smegmatis* AS strain.

### *M. smegmatis* AS strain induced by tetracycline had morphological changes

**Figure 3:**
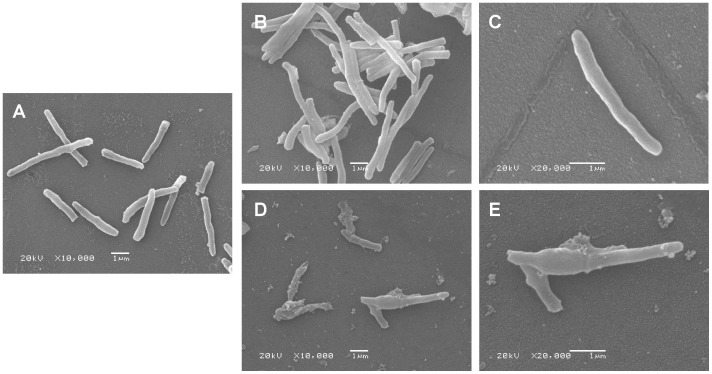
Scanning electron micrographs of wild type mc^2^155 strain and *M. smegmatis* AS strain. Bacterial cells with or without tetracycline were incubated at 37°C for 36 h and harvested respectively for SEM (A–E). A. wild type mc^2^155 strain induced with 20 ng/ml tetracycline (10000×); B, C. *M. smegmatis* AS strain without tetracycline (10000× and 20000×); D, E. *M. smegmatis* AS strain with 20 ng/ml tetracycline (10000× and 20000×).

**Figure 4:**
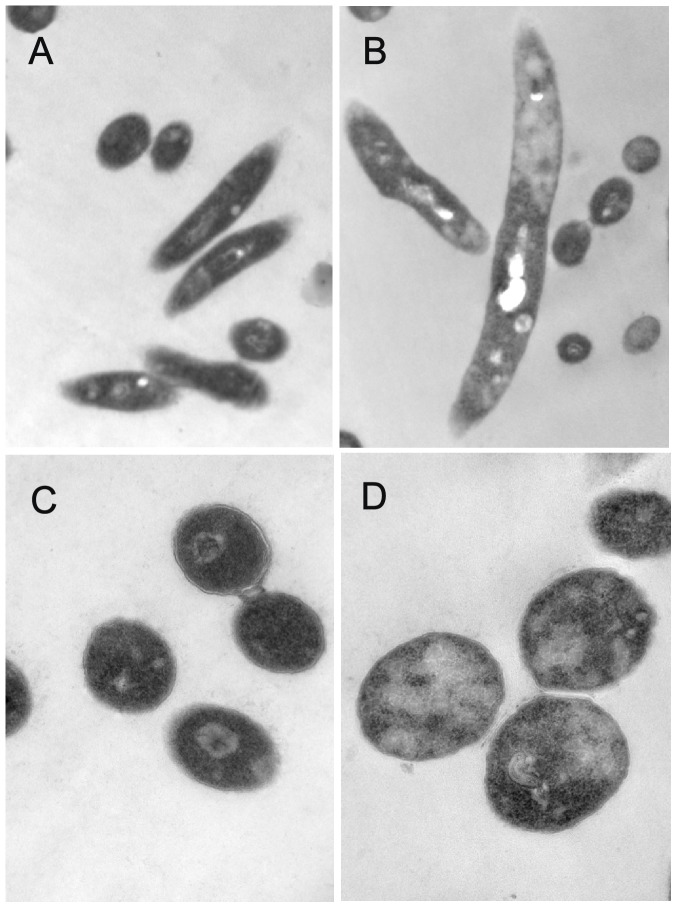
Transmission electron micrographs of wild type mc^2^155 strain and *M. smegmatis* AS strain. Bacterial cells with or without tetracycline were incubated at 37°C for 36 h and harvested respectively TEM observation (A–D). A, C. *M. smegmatis* AS strain without tetracycline (15000× and 60000×); B, D. *M. smegmatis* AS strain induced with 20 ng/ml tetracycline (15000× and 60000×).

**Figure 5:**
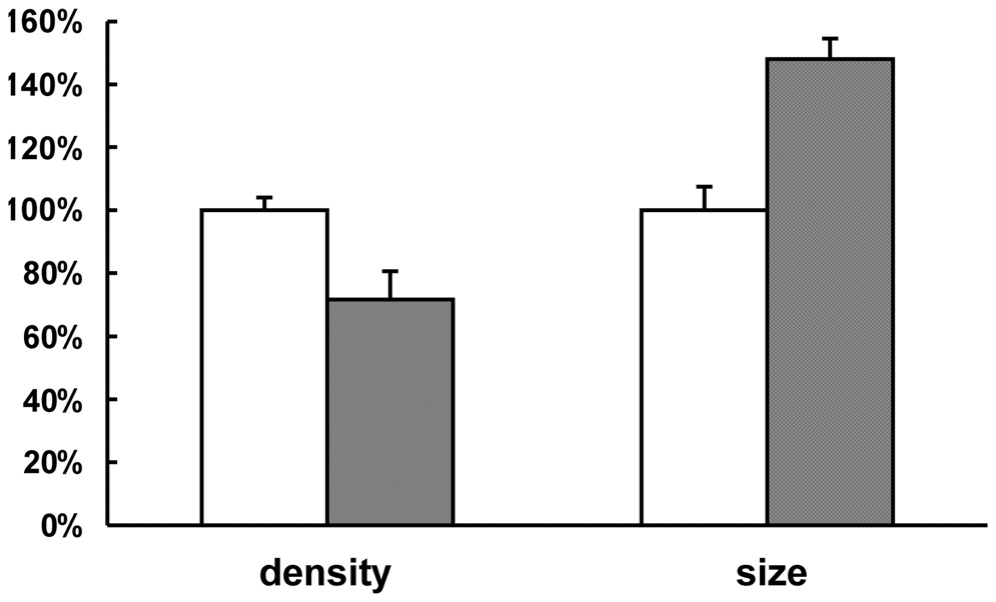
Relative cell size and content density in *M. smegmatis* AS strains. The TEM image of *M. smegmatis* AS strains was analyzed by using Image pro-Plus5.0 soft. The mean value and standard deviation (n = 10) of cell size and content density of uninduced *M. smegmatis* AS strain were measured and the observed values were defined as 100% (white bars). The mean value and standard deviation (n = 8) of induced *M. smegmatis* AS strain were also indicated. Compared with uninduced strain, the cell size in induced *M. smegmatis* AS strain was 0.5 times larger, however, the cell content density was descended by almost 30% (gray bars).

To identify if a decrease in GlmM protein would affect the morphology of *M. smegmatis* AS strain, SEM and TEM were performed. As the images shown, both wild type mc^2^155 strain with 20 ng/ml tetracycline (Fig. 3A) and *M. smegmatis* AS strain without tetracycline (Fig. 3B, 3C) had a rod-like shape and a smooth cell surface similar to wild type mc^2^155 strain [18]. However, there were obvious morphological changes in induced *M. smegmatis* AS strain (Fig. 3D, 3E) compared to wild type mc^2^155 strain. The morphology of *M. smegmatis* AS strain with 20 ng/ml tetracycline exhibited an irregular appearance with a wrinkled cellular surface and an enlarged shape. TEM observation showed that *M. smegmatis* AS strain induced with 20 ng/ml tetracycline appeared to a bigger size and a lower density of cell contents compared to uninduced AS strain (Fig. 4 and Fig. 5). These results indicated that insufficient of GlmM protein lead a morphological changes in mycobacteria.

### GlmM protein affects the biofilm formation in *M. smegmatis*

**Figure 6:**
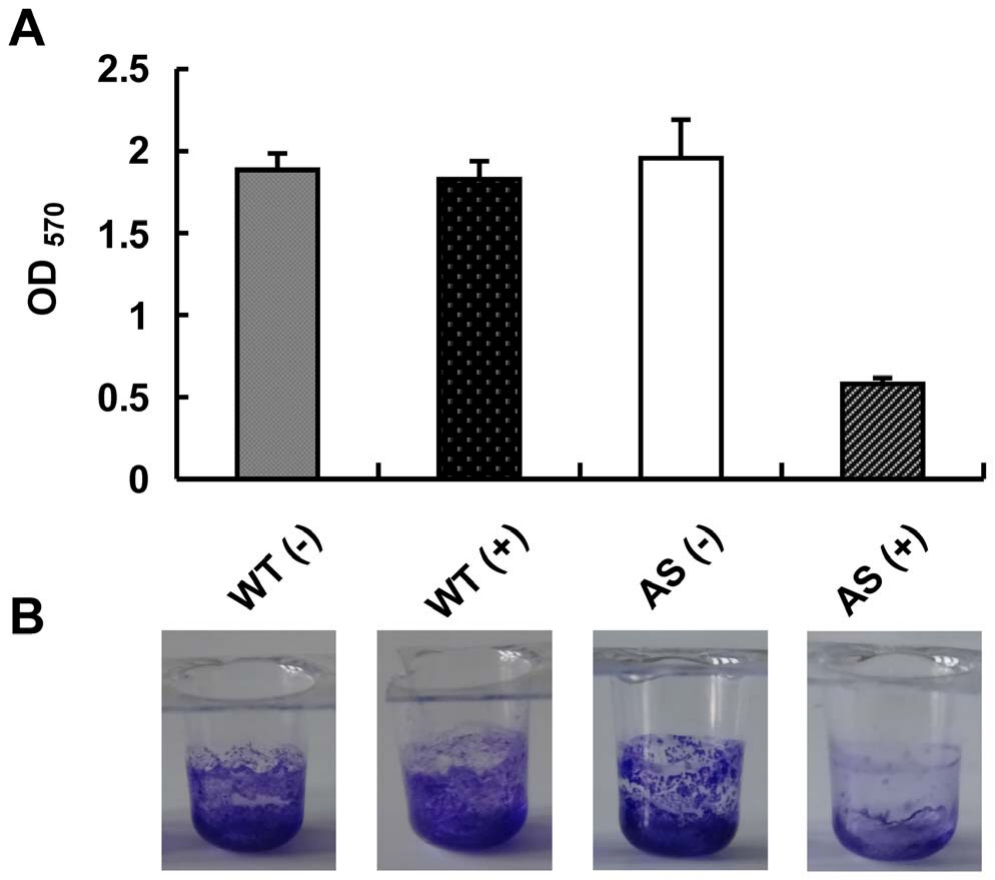
Biofilm formation in wild type mc^2^155 strain and *M. smegmatis* AS strain. Wild type mc^2^155 strain and *M. smegmatis* AS strain were incubated in modified M63 media with or without tetracycline in a polystyrene microtiter plate at 30°C for 5 days without disturbance. Crystal violet stained polystyrene wells (B) and the estimations of corresponding crystal violet uptake (A) were shown for the indicated strain. WT (−). Wild type mc^2^155 strain without tetracycline; WT (+). Wild type mc^2^155 strain induced with 20 ng/ml tetracycline; AS (−). *M. smegmatis* AS strain without tetracycline; AS (+). *M. smegmatis* AS strain induced with 20 ng/ml tetracycline. The experiment was carried out in triplicate and the graph indicates the mean value with standard deviation bars.

To further investigate the roles of GlmM protein, the formation of biofilm in *M. smegmatis* AS strain induced by 20 ng/ml tetracycline was carried out. As shown in Fig 6, the biofilm formation of wild type mc^2^155 strain induced with 20 ng/ml tetracycline was similar as the uninduced strain, however, the *M. smegmatis* AS strain induced with 20 ng/ml tetracycline formed less biofilms compared with the uninduced AS strain (Fig. 6B). This was also confirmed by quantitation of crystal violet staining (Fig. 6A). The result indicated that GlmM had effect on biofilm formation in *M. smegmatis*.

### Insufficient GlmM protein increased the sensitivity to anti-tuberculosis drugs in *M. smegmatis*

To investigate if GlmM protein could affect the sensitivity to anti-tuberculosis drugs in *M. smegmatis*, the MICs of five first-line anti-tuberculosis drugs in wild type mc^2^155 strain and *M. smegmatis* AS strain were determined. As shown in Table 2, the *M. smegmatis* AS strain induced with 20 ng/ml tetracycline was more sensitive to INH and EMB than uninduced AS strain. However, there was no significant difference of antimicrobial susceptibility to STR, PZA and RMF between induced and uninduced AS strains. In addition, all the MICs of anti-tuberculosis drugs to wild type mc^2^155 strain with or without tetracycline were similar to uninduced *M. smegmatis* AS strain.

**Table 2:** MICs of anti-tuberculosis drugs in wild type mc^2^155 strainand *M. smegmatis* AS strain.

	MICs (µg/ml)				
Strains	INH	EMB	RMF	STR	PZA
WT (−)	3.33±1.44	0.26±0.09	0.03±0.00	0.27±0.12	20.80±9.01
WT (+)	2.50±0.00	0.26±0.09	0.03±0.01	0.20±0.00	20.80±9.01
AS (−)	2.08±0.72	0.26±0.09	0.04±0.02	0.20±0.00	20.80±9.01
AS (+)	0.26±0.09	0.02±0.02	0.03±0.01	0.20±0.00	20.80±9.01

WT (−). Wild type mc^2^155 strain without tetracycline; WT (+). Wild type mc^2^155 strain induced with 20 ng/ml tetracycline; AS (−). *M. smegmatis* AS strain without tetracycline; AS (+). *M. smegmatis* AS strain induced with 20 ng/ml tetracycline. The experiment was carried out in triplicate, the mean value and standard deviation were indicated.

## Discussion

The *M. tuberculosis* has a unique cell wall structure, which is essential for bacterial viability. The enzymes involved in different biosynthetic pathways of cell wall are considerable attractive for the development of anti-tuberculosis drugs. In our previous study, GlmM had been identified as phosphoglucosamine mutase to catalyze epimerization between glucosamine-6-phosphate and glucosamine-1-phosphate during the biosynthesis of UDP-GlcNAc, which is a crucial material for cell wall. GlmM is essential for mycobacteria growth, which means it is a potential target for anti-tuberculosis drugs [12]. Here, the biological functions of GlmM protein were investigated.

*M. smegmatis* is commonly used in work on the mycobacterium species due to its fast growing and non-pathogenic. Furthermore, its proteins have high homology with *M. tuberculosis* and it also shares the same cell wall structure of *M. tuberculosis*. These properties make *M. smegmatis* a good model organism for pathogenic *M. tuberculosis*. In addition, the bioinformatics analysis data showed that *M. smegmatis* GlmM protein was high homology with *M. tuberculosis* GlmM protein, which shares 79% identities to each other. Furthermore, it had proved that *glmM* gene was essential for the growth of both *M. tuberculosis* [19] and *M. smegmatis* [12]. For these reasons, *M. smegmatis* was used as a surrogate host for *M. tuberculosis*.

To facilitate the analysis of *glmM* gene functions in mycobacteria, a tetracycline-inducible vector pMind was used to construct an expression strain of *glmM* antisense RNA expression. Different lengths of antisense RNAs were tested in our lab. There was little impact on GlmM protein expression by using full length of *glmM* antisense RNA, however, *glmM* antisense mRNAs with 200, 300 and 420 bases bind with the 5′ end of *glmM* mRNA gave a good and similar effect on inhibition of GlmM protein (data not shown). In this study, *glmM* antisense RNA with 420 bases was chosen for all the experiments. In addition, a blast of the *glmM* antisense RNA fragment (420 bases) against the *M. smegmatis* genome was performed to check its specificity to the *glmM* gene. The results showed that this *glmM* antisense RNA fragment was specifically matched with the *glmM* gene in *M. smegmatis*, which indicated that the *glmM* mRNA was blocked by this antisense RNA fragment for the biosynthesis of GlmM protein.

We had recently constructed a conditional *M. smegmatis glmM* gene knockout strain and confirmed that *glmM* gene was essential for the growth of *M. smegmatis* [12]. However, the further studies of *glmM* gene functions would be hampered by the lethal phenotype of *M. smegmatis glmM* gene knockout strain. In order to get more information of *glmM* gene functions in mycobacteria, a gene knockdown strain (*M. smegmatis* AS strain) was constructed here. The *M. smegmatis* AS strain was induced by tetracycline and the induction was “dose dependent”. The growth of *M. smegmatis* AS strain was almost suppressed with 50 ng/ml tetracycline. However, to ensure that we could get a sufficient amount of living cells for further analysis, only tetracycline at low concentration could be chosen as the inducer for *M. smegmatis* AS strain. In our study, the *M. smegmatis* AS strain was induced with 20 ng/ml tetracycline for 36 h. Under this condition, the GlmM protein expression in AS strain was fallen by about 70%, which was sufficient to cause the decreased rate of cell growth and the visible morphology changes. Furthermore, a certain amount of living cells could be collected. More importantly, there was no effect on normal growth when wild type *M. smegmatis* induced with 20 ng/ml tetracycline. All of these demonstrated that the *M. smegmatis* AS strain could be used for studying the roles of *glmM* gene.

In the study we found that the inductive effect became weaker after induction by tetracycline for 36 h, there were two possible reasons to explain this phenomenon. One was the stability of tetracycline, which was an important factor for sustained induction of *glmM* antisense mRNA expression. Tetracycline is unstable and easily degraded by both acidic and alkaline environments. We found the pH value of culture was slightly raised due to metabolic products of bacteria, which might cause a degradation of tetracycline. Another possible explanation is that at the initial of cell growth, the GlmM protein expressed in tetracycline-induced *M. smegmatis* AS strain was staying in a lower level, which could not maintain a normal growth. However, with the extended culture time, the GlmM protein would gradually accumulate to a certain level due to degradation of tetracycline, therefore, the cell growth would be restored.

The morphology of *M. smegmatis glmM* gene knockdown strain was compared with that of *glmM* gene knockout strain, *M. smegmatis* LS2 [12]. After induced with 20 ng/ml tetracycline for 36 h, the morphology of *M. smegmatis* AS strain showed a wrinkled cellular surface and an enlargement shape, but no obvious cell lysis was observed as *M. smegmatis* LS2. This indicated that the expression of GlmM protein in *M. smegmatis glmM* gene knockdown strain reduced significantly, but not at lethal level. We noticed that cell debris were observed in *M. smegmatis* AS strain which were induced with 20 ng/ml tetracycline by SEM, but no similar phenomenon was observed in uninduced AS strain. Another notable phenomenon was that we could not obtain a compact pellet when the cells of induced *M. smegmatis* AS strain were centrifuged. These details gave us a hint that the cell of induced *M. smegmatis* AS strain was abnormal. Considering the irregular cellular surface and the reduced cell contents of induced *M. smegmatis* AS strain, we speculated that insufficient GlmM activity destroyed the integrity of cell wall, increased the permeability and made the cell contents spilling.

Biofilm in bacteria can significantly enhance their resistance to antibiotics. Previous studies had proved that *glmM* mutation would reduce biofilm formation in *Streptococcus mutans* and *Streptococcus gordonii* [20], [21]. Here, the biofilm formation in *M. smegmatis* AS strain was investigated. We found that the biofilm formation was reduced when *M. smegmatis* AS strain was induced with 20 ng/ml tetracycline, indicated that *glmM* gene had effect on the biofilm formation of *M. smegmatis*. For the development of biofilm formation in *M. smegmatis*, Recht *et al*. had showed that the glycopeptidolipids, which was known to be present in the outermost layer of the envelope, was important for initial surface attachment during biofilm formation [22]. Ojha *et al.* found that a short chain mycolic acid (C_56_–C_68_) played an important role in the development of biofilm architecture in *M. smegmatis* [23]. Although GlmM enzyme was not involved in the biosynthesis process of mycolic acid and glycopeptidolipids directly, the reduced GlmM expression might impair the cell wall integrity which influenced covalent attachment of mycolic acid to arabinogalactan and anchoring of glycopeptidolipids and subsequently reduced the biofilm formation.

Shimazu *et al*. and Jolly *et al.* had reported that *glmM* mutations in *Streptococcus gordonii* and *Streptococcus aureus* result in reduced methicillin resistance [20], [24]. The susceptibility test for antimicrobials was also carried out in this study. The results showed that *M. smegmatis* AS strain induced with 20 ng/ml tetracycline was more sensitive to INH and EMB than uninduced AS strain. Interestingly, all of these drugs including INH, EMB and methicillin target the bacterial cell wall, which indicated that inhibition of GlmM protein could facilitate the efficacy of anti-tuberculosis drugs which target on cell wall synthesis. In addition, we should note that the MIC to PZA was determined at a neutral pH, which was not the optimum pH (pH 5.5) for PZA activity [25]. Generally, *M. smegmatis* grow in the neutral pH. Furthermore, the tetracycline is easily degraded in the acid condition. To test the impact of pH on *M. smegmatis* growth and tetracycline stability, additional experiment was performed as follow: *M. smegmatis* AS strain was inoculated in LB broth containing 0.05% Tween 80 at pH 5.5, and tetracycline at 0 ng/ml and 20 ng/ml was added respectively. We found that the growth of M. smegmatis AS strain was slowly in the acid medium, and no difference of the growth rate was detected between two groups (data not shown). The results demonstrated that the acidic pH would slow down the growth of *M. smegmatis* and decrease the stability of tetracycline. Considering the above factors, the MIC experiment was performed at a neutral pH.

These results provide a new insight on GlmM functions in mycobacteria. Due to the significant role in biofilm formation and sensitivity to antibiotics, GlmM is expected to be a potential target for development of new anti-tuberculosis drug.
